# Combination of bulk RNA sequencing and scRNA sequencing uncover the molecular characteristics of MAPK signaling in kidney renal clear cell carcinoma

**DOI:** 10.18632/aging.205436

**Published:** 2024-01-12

**Authors:** Xiunan Li, Hewen Guan, Chuanyu Ma, Yunfei Dai, Ji Su, Xu Chen, Qihang Yuan, Jianbo Wang

**Affiliations:** 1 Department of Urology, The First Affiliated Hospital of Dalian Medical University, Dalian, Liaoning, China; 2 Department of Dermatology, First Affiliated Hospital of Dalian Medical University, Dalian, Liaoning, China; 3 Department of General Surgery, First Affiliated Hospital of Dalian Medical University, Dalian, Liaoning, China; 4 Department of Urology, Central Hospital of Benxi, Benxi, Liaoning, China

**Keywords:** bulk RNA sequencing, single-cell RNA sequencing, molecular characteristics, MAPK signaling, kidney renal clear cell carcinoma

## Abstract

The MAPK signaling pathway significantly impacts cancer progression and resistance; however, its functions remain incompletely assessed across various cancers, particularly in kidney renal clear cell carcinoma (KIRC). Therefore, there is an urgent need for comprehensive pan-cancer investigations of MAPK signaling, particularly within the context of KIRC. In this research, we obtained TCGA pan-cancer multi-omics data and conducted a comprehensive analysis of the genomic and transcriptomic characteristics of the MAPK signaling pathway. For in-depth investigation in KIRC, status of MAPK pathway was quantitatively estimated by ssGSEA and Ward algorithm was utilized for cluster analysis. Molecular characteristics and clinical prognoses of KIRC patients with distinct MAPK activities were comprehensively explored using a series of bioinformatics algorithms. Subsequently, a combination of LASSO and COX regression analyses were utilized sequentially to construct a MAPK-related signature to help identify the risk level of each sample. Patients in the C1 subtype exhibited relatively higher levels of MAPK signaling activity, which were associated with abundant immune cell infiltration and favorable clinical outcomes. Single-cell RNA sequencing (scRNA-seq) analysis of KIRC samples identified seven distinct cell types, and endothelial cells in tumor tissues had obviously higher MAPK scores than normal tissues. The immunohistochemistry results indicated the reduced expression levels of PAPSS1, MAP3K11, and SPRED1 in KIRC samples. In conclusion, our study represents the first integration of bulk RNA sequencing and single-cell RNA sequencing to elucidate the molecular characteristics of MAPK signaling in KIRC, providing a solid foundation for precision oncology.

## INTRODUCTION

Originating from renal tubular epithelial cells, renal cell carcinoma is a malignant tumor accounts for nearly 80% of renal malignancies [1]. Kidney renal clear cell carcinoma (KIRC), with rising incidence and dismal prognosis, is the most common type of renal cell carcinoma [2]. Unfortunately, KIRC is intrinsically resistant to radiation and chemotherapy and only a limited number of treatments such as targeted therapy can be taken [3]. It is not only urgent but also necessary to explore sensitive biomarkers and treatment options for KIRC patients to improve the prognosis.

The mitogen-activated protein kinase (MAPK) signaling pathway is significant in inter- and intra-cellular communication, which affects the cellular processes such as cell proliferation and differentiation [4]. Through three capital kinases: mitogen-activated protein kinase (MAPK), mitogen-activated protein kinase kinase, and mitogen-activated protein kinase kinase kinase, MAPK pathway transforms external stimuli into cellular responses [5]. Current studies concentrating on the influence of the MAPK pathway on the development and metastasis of cancer indicate that the signaling pathway actually acts as a regulator in many cancers such as colorectal cancer and non-small cell lung cancer [5–8]. In addition, MAPK signaling was identified as a potential mechanism to regulate and tumor resistance and drug sensitivity [9].

However, few in-depth researches are reported to reveal the influence of MAPK pathway in KIRC and other cancers. In this research, the roles of MAPK pathway in pan-cancer are summarized and the relation between MAPK pathway and KIRC is purposefully explored. First, 3 different KIRC subtypes with different MAPK signaling activity were identified. Subsequently, the influences of MAPK signaling on the metabolism-related pathways, immune-related pathways, immune response, and ICG expression were investigated. Then 11 hub genes (i.e., SPRED3, ACTB, ARAF, MAP3K11, PAPSS1, TLN1, CALM1, AGK, MAP2K2, MAPK1, SPRED1), selected from all the MAPK-related genes, were utilized to construct a prognostic signature. The signature helped to distinguish KIRC samples with different risk levels. Endothelial cells in tumor tissues had obviously higher MAPK scores than normal tissues. The immunohistochemistry indicated that the PAPSS1, MAP3K11, and SPRED1 showed lower IHC score in KIRC compared with para-cancer samples. All the discrepancies between high- and low-risk subgroups were explored and all these could be potential therapy targets in KIRC.

## MATERIALS AND METHODS

### Data acquisition

In the current study, the pan-cancer mRNA expression, single nucleotide variation (SNV), copy number variation (CNV), DNA methylation data were obtained from The Cancer Genome Atlas (TCGA) database (https://portal.gdc.cancer.gov/). In addition, the clinical parameters of KIRC were downloaded simultaneously. The KIRC transcriptome profiles were also searched in the ArrayExpress database (https://www.ebi.ac.uk/arrayexpress/). The Molecular Signatures Database (MSigDB) were searched to obtain the MAPK pathway and other signaling pathways (https://www.gsea-msigdb.org/gsea/msigdb/human/geneset/REACTOME_ONCOGENIC_MAPK_SIGNALING.html?keywords=REACTOME_ONCOGENIC_MAPK_SIGNALING). Immune checkpoint genes (ICGs) were summarized according to the review [10].

### Comprehensive assessment of MAPK pathway in pan-cancer

Recent studies indicated that MAPK pathway affect the biological behavior and prognosis of malignancies and targeting MAPK pathway may be a novel perspective for cancer therapy [9, 11–14]. Nonetheless, the prognostic value, expression level, CNV, SNV and methylation of key genes in MAPK pathway in pan-cancer are reported dispersedly and sparsely. Subsequently, comprehensive assessments of genes regulating MAPK pathway were conducted in pan-cancer. First, the expression of MAPK genes in each cancer were compared with those in corresponding normal tissues and each fold change was calculated respectively [15]. Then the CNV gain, CNV loss, and SNV were summarized and presented utilizing heatmap. In addition, the estimations of DNA methylation of MAPK genes in pan-cancer were conducted by comparing with corresponding normal samples [16].

### MAPK-based cluster analysis in KIRC

Due to the potential role of MAPK signaling pathway in KIRC, the activation of the pathway was assessed for the first step. With the expression levels of the MAPK genes in KIRC, the status of MAPK pathway was quantitatively estimated by ssGSEA. After acquiring the MAPK score, KIRC samples were grouped into cluster1 with MAPK-active status, cluster2 with MAPK-normal status, and cluster3 with MAPK-inactive status through cluster analysis by Ward algorithm. Then the violin plots of MAPK scores were constructed and the survival curves were plotted to explore the survival discrepancies among these three clusters. Following a methodology similar to previous studies [17, 18], we curated a selection of classical immune pathways, metabolic pathways, and cell death pathways. Initially, we computed pathway scores using the “gsva” package, providing a measure of each pathway’s activity. Subsequently, we consolidated these scores and visually represented them in a heatmap format. Statistical analyses were carried out using the “kruskal.test” function in R.

For investigation of the tumor microenvironment (TME) in the three clusters, the “ESTIMATE” package in R and various immune-related algorithms including TIMER, QUANTISEQ, MCPCOUNTER, XCELL, EPIC, and CIBERSORT were utilized for further analyses [19]. Additionally, the expression levels of common immune checkpoint genes were compared in the three clusters utilizing Kruskal-Wallis test. By applying the ssGSEA, the immune response was estimated. Next, we delved into the correlations between MAPK signaling and the infiltration of immune cells, and the findings were visually presented in a heatmap.

For further exploration about the discrepancies of the drug sensitivity in the three clusters, the Genomics of Drug Sensitivity in Cancer database (GDSC; https://www.cancerRxgene.org) was utilized to predict therapy response. The GDSC database linked drug sensitivity to genomic data then the IC50 of the samples were obtained. Of note, a lower IC50 suggests that the cancer cells are more sensitive to the compound.

### Construction and validation of a MAPK-related signature

In view of the significant role of MAPK pathway in KIRC, then all the MAPK genes were utilized to construct a signature to help distinguish KIRC samples. To ensure adequate validation, all samples in TCGA were randomly divided into a training set and a test set 1 at a nearly 1:1 ratio. In addition, all samples from TCGA and ArrayExpress were defined as test2 cohort and test3 cohort, respectively.

In train cohort, LASSO and Cox regression analysis were utilized to identify the hub genes and construct a MAPK-related signature (MAPKS) to help distinguish KIRC samples. After identifying the MAPKS, the “predict” function in R was utilized to calculate the risk score. Then all the samples were grouped into high- and low-risk subgroups based on the median risk score in train cohort. In the four cohorts, the following discrepancies between high- and low-risk subgroups were investigated for comprehensive validation: (1) the survival analysis was performed to identify the survival discrepancy; (2) the receiver-operating characteristic (ROC) curves were utilized to determine the diagnostic value of MAPKS; (3) “ESTIMATE” package in R was utilized to assess the tumor microenvironment [20]; (4) TIMER, QUANTISEQ, and many other algorithms introduced above were utilized to assess the immune response in the TME [21]; (5) the expression levels of ICGs in high- and low-risk subgroups were compared.

### The estimation of MAPK pathway and gene expression of MAPKS genes on the basis of scRNA-seq data

KIRC scRNA-seq data, GSE159115, was obtained from the GEO database. The scRNA-seq data of KIRC were analyzed based on the standard protocols of Seurat [22]. Those cells with less than 200 or more than 7000 count features were removed. In addition, cells with mitochondrial RNA percentage > 10 were also excluded. Then the data were normalized, scaled and processed for PCA analysis. The Harmony package was utilized to remove the batch effect. The “FindClusters” function was used to cluster cells at an appropriate resolution. The t-SNE was utilized to visualize the data. Based on the typical cell-type markers, all the subpopulations were annotated. The activity of MAPK pathway was estimated utilizing five well-known algorithms (i.e. AUCell, UCell, singscore, Add, and GSVA). Of note, the scores from the five algorithms mentioned above were summed to obtain a total score, which we referred to as “Scoring”. We employed the “wilcox.test” to compare pathway activities between KIRC and normal samples at the single-cell resolution.

### Identification of hub genes in the occurrence of KIRC

On the basis of the genes in the MAPKS, in-depth exploration was conducted to identify hub genes in KIRC. First, the GEPIA online server was utilized to compare the mRNA expression of all the module genes in our signature. Then the relationship between the expression of each gene and tumor stage in KIRC was explored. In addition, the Biomarker Exploration for Solid Tumors (BEST) web server was used for further investigation of the relationship between the expression of each gene and tumor grade.

### External validation of hub genes in MAPKS based on the tissue microarray and immunohistochemistry (IHC)

Human KIRC tissue chips were purchased from Zhuoli Biotechnology Co., Shanghai, China. First, EDTA was used to extract antigens after tissue dewaxing. After placing with the primary antibodies, the tissue sections were incubated with the secondary antibodies. Then the diaminoaniline staining was made and hematoxylin was utilized to re-stain. Finally, the IHC morphology of 80 KIRC samples and 80 normal samples were completed. The IHC scores were analyzed by two independent pathologists based on the staining intensity and the percentage of positive-stained cells intensity.

### Availability of data and materials

The datasets analyzed in this work may be found in the Supplementary Materials or contact with the corresponding author.

## RESULTS

### Changes of mRNA expression, CNV, SNV, and methylation of MAPK-related genes in pan-cancer

**Figure 1:**
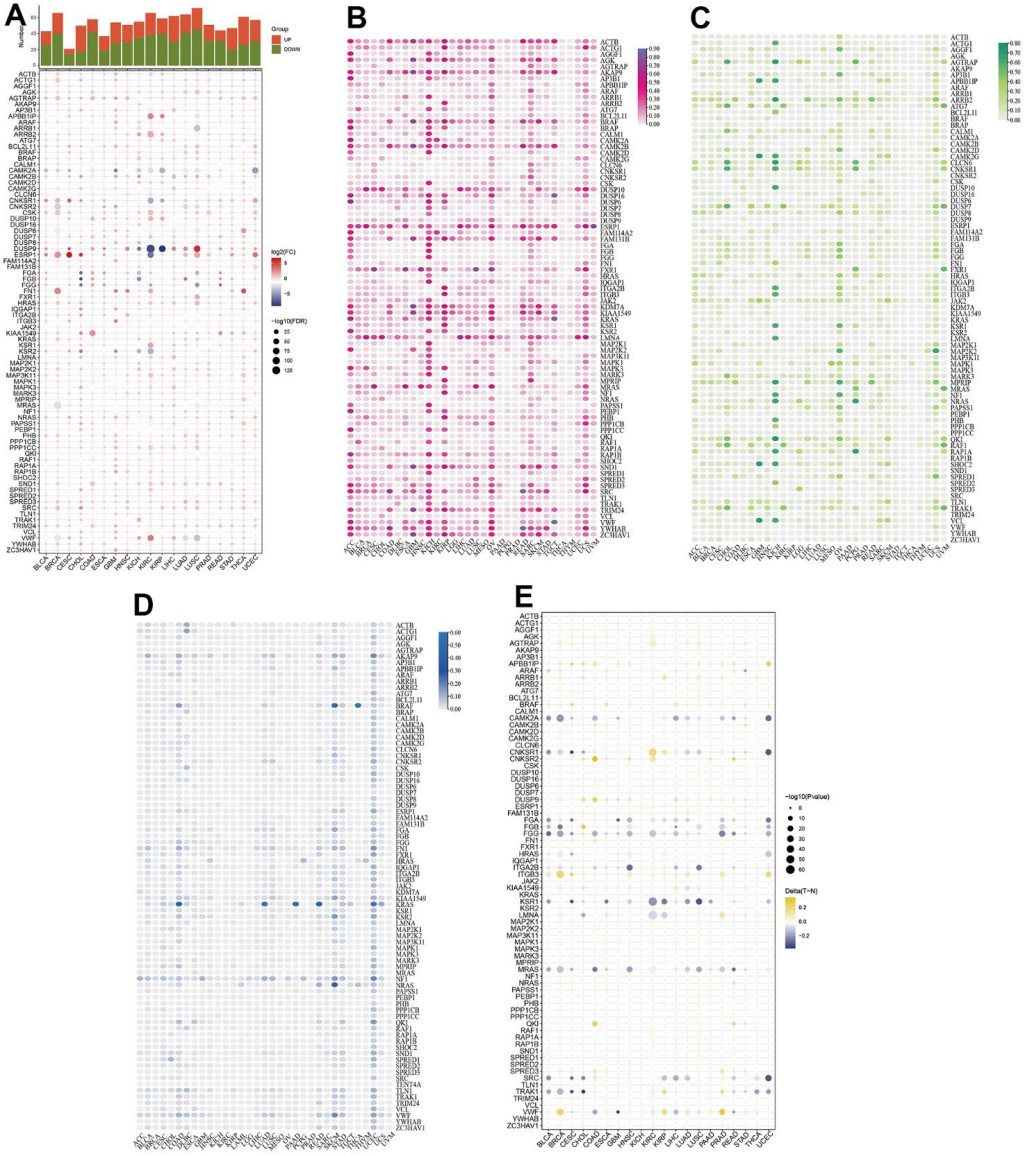
**Comprehensive assessment of MAPK pathway in pan-cancer.** (**A**) mRNA expression of MAPK-related genes. (**B**) CNV gain of MAPK-related genes. (**C**) CNV loss of MAPK-related genes. (**D**) SNV frequency of MAPK-related genes. (**E**) DNA methylation of MAPK-related genes.

First, the expression levels of MAPK-related genes were summarized in pan-cancer. It was shown in Figure 1A that obvious up-regulation of ESRP1 existed in CESC, perceptible up-regulation of DUSP9 existed in LUSC, while significant down-regulation of DUSP9 existed in KIRC and KIRP. As a major influence in the gene expression levels, the CNV gain and CNV loss needed to be paid more attention to. As shown in Figure 1B, the pinker the color, the higher the CNV gain frequency. In KICH, the number of MAPK genes with high CNV gain frequency is maximum. And in KIRC, CAMK2A and FAM114A2 had obviously high CNV gain frequency. As for the CNV loss frequency in the Figure 1C, the number of MAPK genes with high CNV loss frequency is also maximum in KICH. And in KIRC, ATG7, RAF1, and TRAK1 had high CNV loss frequency. In addition, increasing SNV frequency of KRAS existed in COAD, LUAD, PAAD, READ, and UCEC (Figure 1D). As for the DNA methylation, the cancers and the corresponding genes with hypermethylation were as follows: BRCA: VWF and ITGB3; COAD: QKI, DUSP9, and CNKSR2; KIRC: CNKSR1; PRAD: VWF, LMNA, and ITGB3; UCEC: ITGB3 and APBBAIP (Figure 1E).

### MAPK-based cluster analysis in KIRC

**Figure 2:**
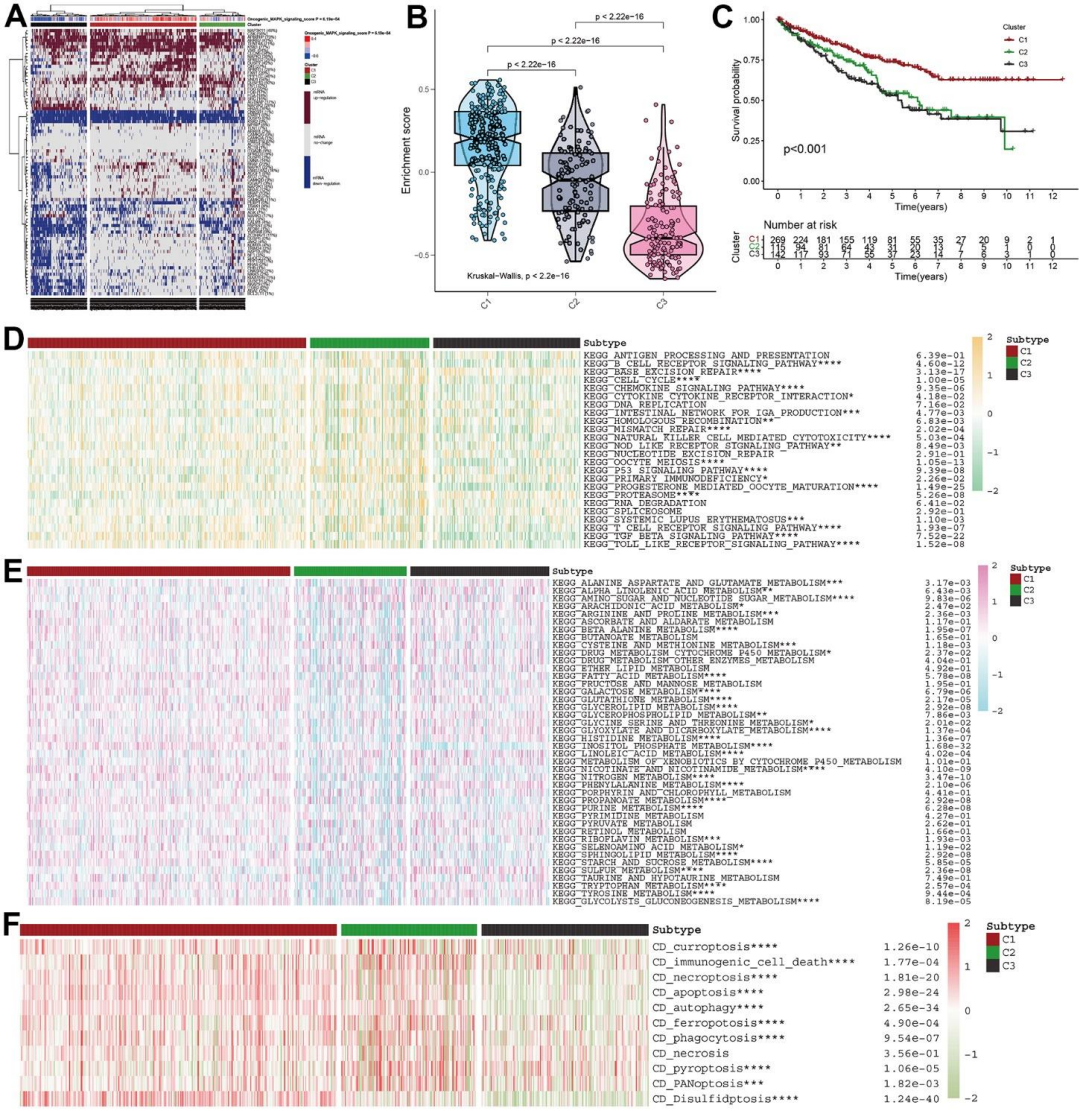
**MAPK-based cluster analysis in KIRC.** (**A**) Three clusters were obtained and displayed by the heatmap. (**B**) The violin plot showing the enrichment scores of these three clusters. (**C**) The distinct of survival probability in these three clusters. (**D**) The discrepancies of metabolism pathway activity in the three clusters. (**E**) The discrepancies of immune pathway activity in the three clusters. (**F**) The discrepancies of cell death status in the three clusters.

For further demonstration of the role of MAPK pathway in KIRC, all KIRC samples were classified into three clusters (Figure 2A). Subsequently, the MAPK scores in these three clusters were compared and the violin plots in Figure 2B indicated that the cluster1 was MAPK-active cluster, cluster2 was the MAPK-normal cluster while C3 was the MAPK-inactive cluster (Enrichment score: C1>C2>C3, *p*<0.01). The survival analysis indicated that the prognosis of cluster 1 was better than that of cluster 2 and the prognosis of cluster 3 was worse than that of cluster 2. All the discrepancies of the survival rate in the three clusters were statistical (Figure 2C).

### MAPK-based discrepancies in signaling activity and cell death status

With different activity of MAPK signaling, these three clusters had different metabolism signaling, immune signaling. All the statistical discrepancies were exhibited in the form of heatmap. As is shown in Figure 2D, most metabolism signaling had decreasing activity in C2, but AMINO_SUGAR_AND_NUCLEOTIDE_SUGAR_METABOLISM, CYSTEINE_AND_METHIONINE_METABOLISM, and SULFUR_METABOLISM had decreasing activity in C1 and INOSITOL_PHOSPHATE_METABOLISM had decreasing activity in C3. The discrepancies of immune signaling shown in Figure 2E indicated that most immune pathways had decreasing activity in C3 except BASE_EXCISION_REPAIR and PROTEASOME. As for the cell death status shown in Figure 2F, it was indicated that all the types of cell death (i.e., curroptosis, immunogenic cell death, necroptosis, apoptosis, autophagy, ferropotosis, phagocytosis, necrosis, pyroptosis, PANoptosis, disulfidptosis) had decreasing activation in C3.

### MAPK-based discrepancies in immune microenvironment

**Figure 3:**
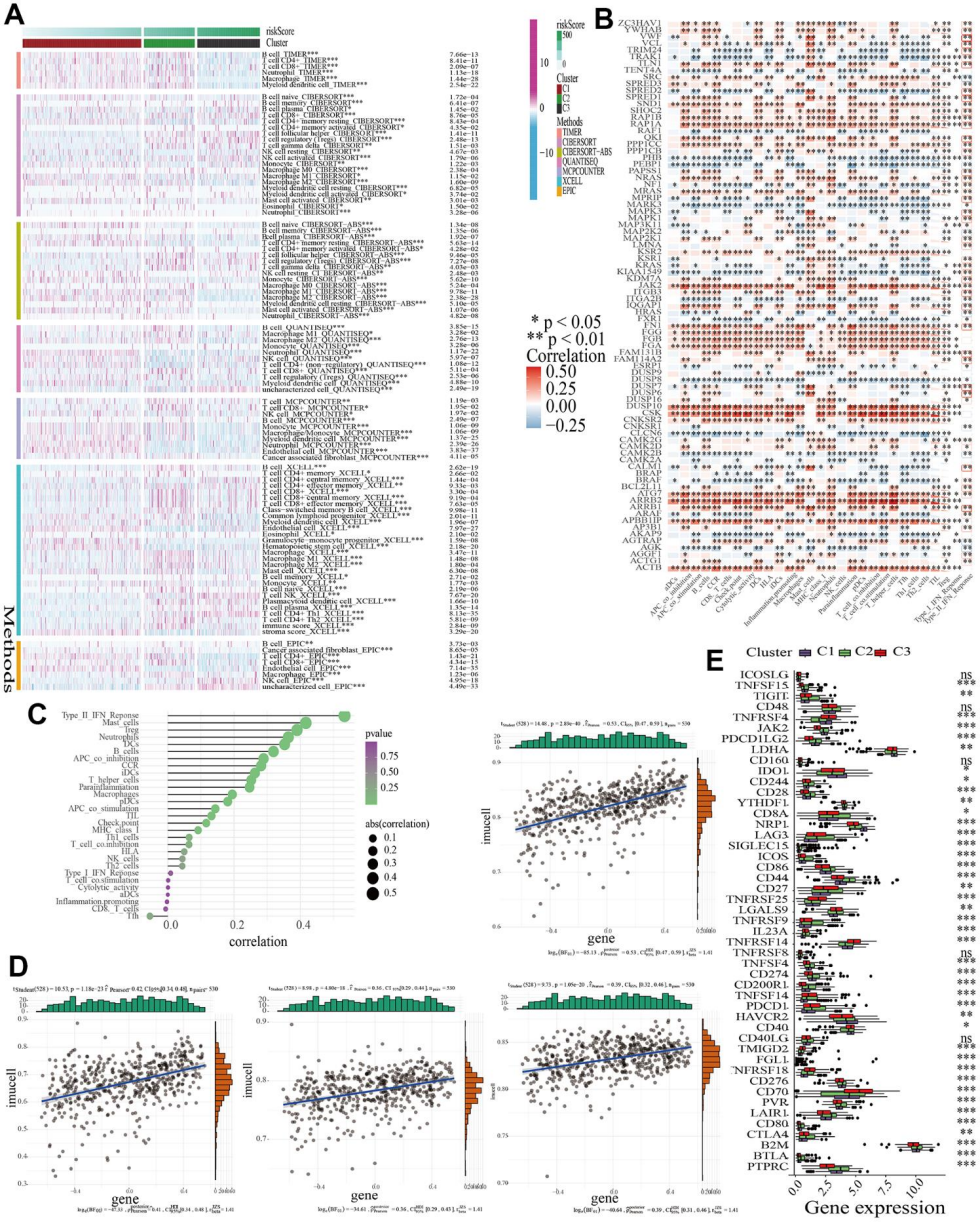
**The discrepancies of TME in the three clusters.** (**A**) A heatmap showing the infiltration of various immune cells. (**B**) The correlations between the expression of MAPK genes and the infiltration of various immune cells. (**C**) The correlations between MAPK score and the infiltration of various immune cells. (**D**) The correlations between MAPK score and the response of mast cell, Treg, neutrophil, and type-II-IFN-Response. (**E**) The discrepancies of ICG expression in the three clusters.

As is known, immune cells are primary components in TME. With various algorithms including TIMER, QUANTISEQ, MCPCOUNTER, XCELL, EPIC, and CIBERSORT, it was found that the infiltration of immune cells differed in the three clusters and most immune cells including B cell, CD4+ T cell, macrophage, neutrophil, and mast cell had lower proportions in C3 (Figure 3A). Further exploration about the correlations between MAPK genes and immune cell infiltration were shown in a heatmap in Figure 3B. The redder the color, the closer the positive correlation. The bluer the color, the closer the negative correlation. As for the correlation between MAPK score and the infiltration of immune cells, it was found that the infiltration of most immune cells except Tfh had positive correlation with MAPK score (Figure 3C). Of note, the responses of mast cell, Treg, neutrophil, and type-II-IFN-Response are positively related to the MAPK score (R>0.3, *p*<0.05) (Figure 3D). Additionally, the immune checkpoint genes had different expression levels in the three clusters. The following genes had lowest expression level in C3: TNFSF15, JAK2, PDCD1LG2, LDHA, CD244, CD28, YTHDF1, NRP1, SIGLEC15, ICOS, CD86, CD44, TNFSF4, CD274, CD200R1, HAVCR2, CD276, PVR, LAIR1, CD80, B2M, BTLA, and PTPRC (*p*<0.05) (Figure 3E).

### MAPK-based drug sensitivity analysis in KIRC

**Figure 4:**
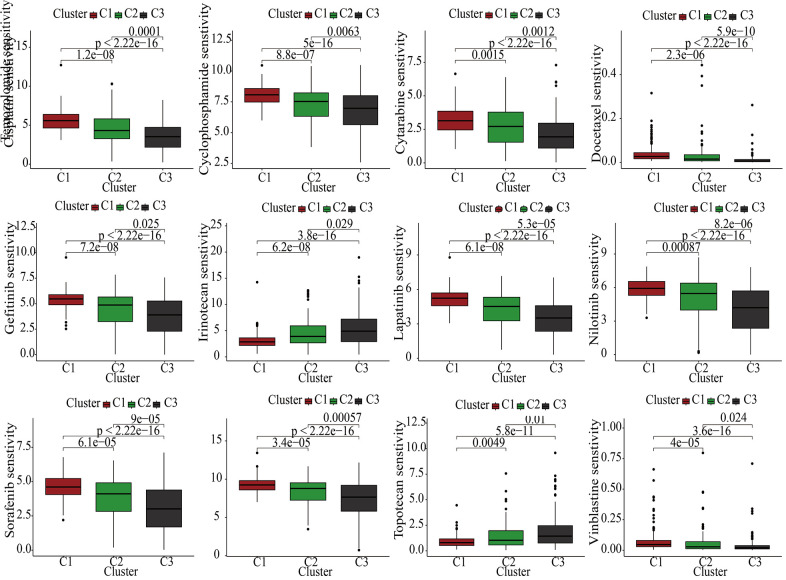
The discrepancies of drug sensitivities in the three clusters.

Currently, the treatment of advanced KIRC mostly depends on molecular targeted drugs. Till now, many types of targeted drugs including sorafenib and sunitinib have been listed in NCCN for a first- or second-line treatment of metastatic kidney cancer [23]. In view of the significant role of the targeted therapy in KIRC, common targeted drugs were taken into consideration for the exploration of the sensitive drugs. The lower the IC50, the more sensitive the compound. For Irinotecan and Topotecan, samples in cluster1 had lowest IC50. For Cisplatin, Cyclophosphamide, Cytarabine, Docetaxel, Gefitinib, Lapatinib, Nilotinib, Sorafenib, Temozolomide, and Vinblastine, samples in cluster3 had lowest IC50 (Figure 4).

### Construction and validation of a MAPK-related signature

**Figure 5:**
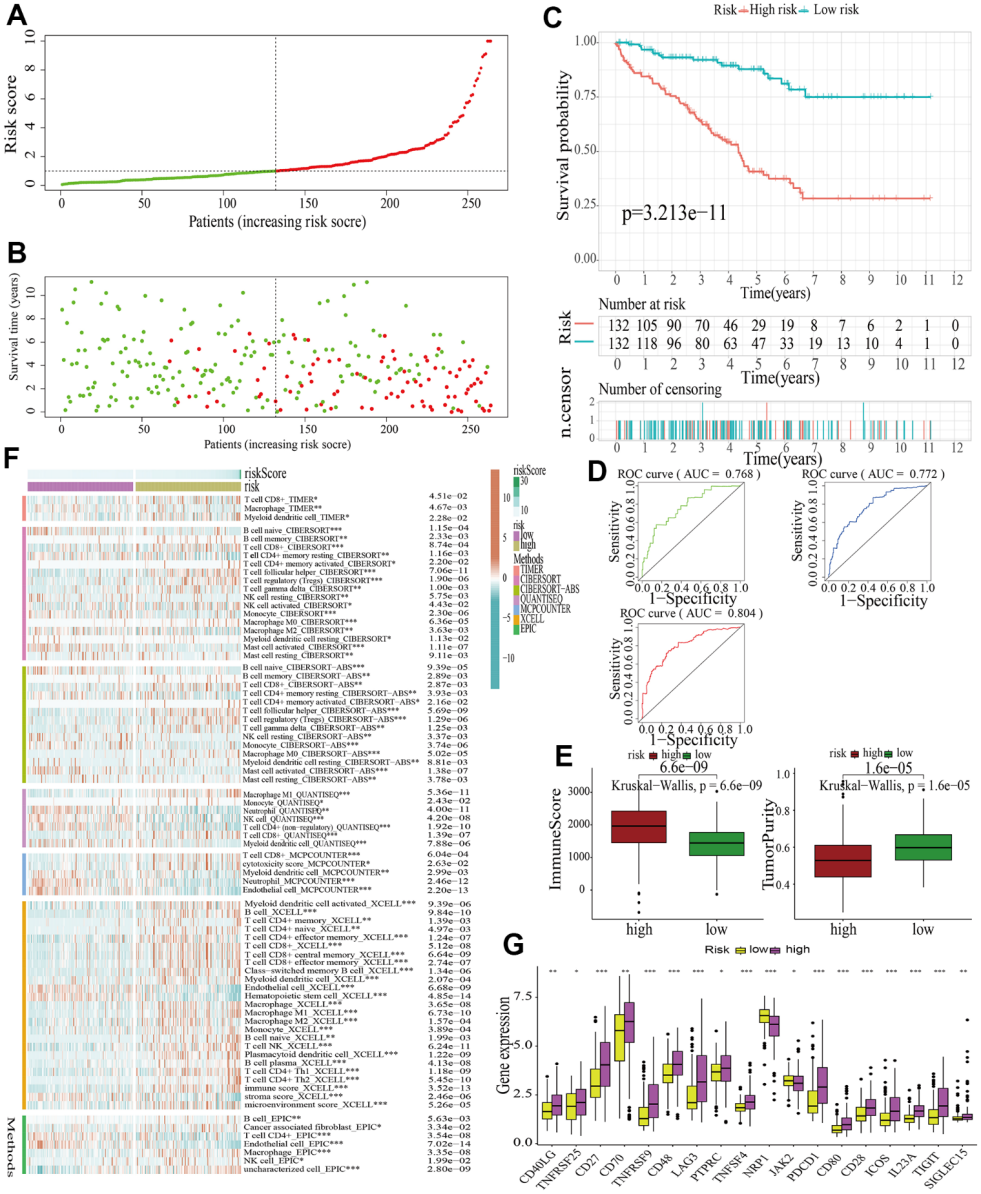
**Identification of a MAPK-related signature in train cohort.** (**A**) The distinguishment of high- and low-risk subgroups on the basis of the median risk score. (**B**) The distributions of the risk score and survival status. (**C**) Survival analysis in train cohort. (**D**) ROC curves of 1-, 3-, and 5-year survival. (**E**) Assessment of TME by “ESTIMATE”. (**F**) The discrepancies of immune response in high- and low-risk subgroups. (**G**) The discrepancies of ICD expression in high- and low-risk subgroups.

Samples in the train cohort were utilized to construct MAPKS. After conducting LASSO regression analysis, univariate Cox regression analysis and multivariate Cox regression analysis, 11 hub genes (i.e., SPRED3, ACTB, ARAF, MAP3K11, PAPSS1, TLN1, CALM1, AGK, MAP2K2, MAPK1, SPRED1) were selected to construct a MAPKS. On the basis of the expression levels of these 11 genes, the risk score of each sample was calculated utilizing the “predict” function in R. Based on the median risk score, samples were divided into high- and low-risk subgroups (Figure 5A). Then the Figure 5B depicted the distributions of the risk score and survival status. Likewise, the survival analysis indicated that patients in high-risk subgroup had lower survival probability (Figure 5C). The AUCs of the ROC curves had values of 0.768, 0.772, and 0.804, respectively, for 1-, 3-, and 5-year survival (Figure 5D). Based on the “ESTIMATE”, it was found that samples in high-risk subgroup had higher immune score but lower tumor purity (Figure 5E). As for immune response in the two subgroups, Treg, Tfh, and gamma delta T cell had higher proportion, while Neutrophil and Endothelial cell had lower proportion in high-risk subgroup in train cohort (Figure 5F). The expression of ICGs in the high- and low-risk subgroups also differed from each other. The expression of most ICGs (i.e., CD40LG, TNFRSF25, CD27, CD70, TNFRSF9, CD48, LAG3, PTPRC, TNFRSF4, PDCD1, CD80, CD28, ICOS, IL23A, TIGIT, SIGLEC15) up-regulated while NRP1 and JAK2 down-regulated in the high-risk subgroup (Figure 5G).

**Figure 6:**
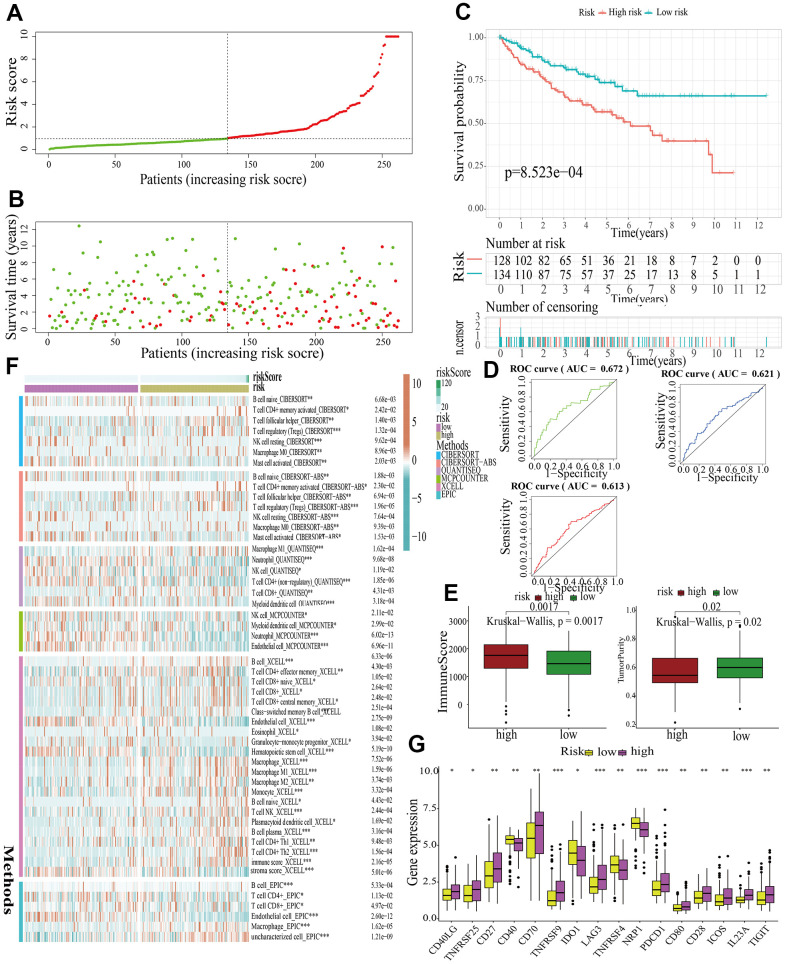
**Internal validation of a MAPK-related signature in test1 cohort.** (**A**) The distinguishment of high- and low-risk subgroups based on the median risk score in train cohort. (**B**) The distributions of the risk score and survival status. (**C**) Survival analysis in test1 cohort. (**D**) ROC curves of 1-, 3-, and 5-year survival. (**E**) Assessment of TME by “ESTIMATE”. (**F**) The discrepancies of immune response in high- and low-risk subgroups. (**G**) The discrepancies of ICD expression in high- and low-risk subgroups.

**Figure 7:**
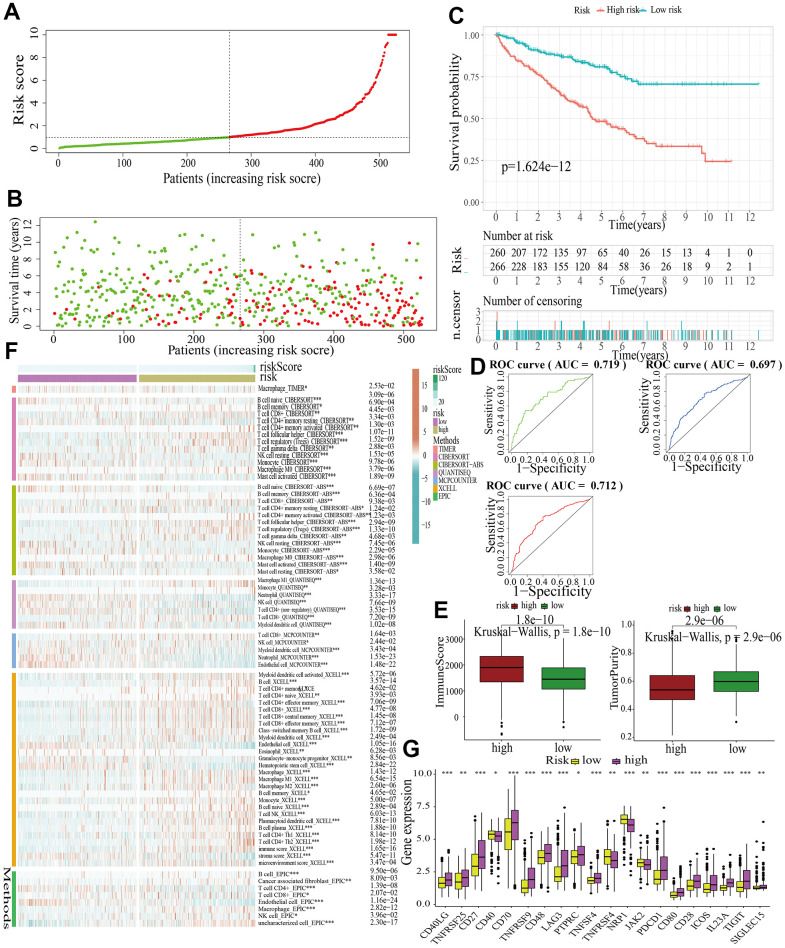
**Internal validation of a MAPK-related signature in test2 cohort.** (**A**) The distinguishment of high- and low-risk subgroups based on the median risk score in train cohort. (**B**) The distributions of the risk score and survival status. (**C**) Survival analysis in test2 cohort. (**D**) ROC curves of 1-, 3-, and 5-year survival. (**E**) Assessment of TME by “ESTIMATE”. (**F**) The discrepancies of immune response in high- and low-risk subgroups. (**G**) The discrepancies of ICD expression in high- and low-risk subgroups.

**Figure 8:**
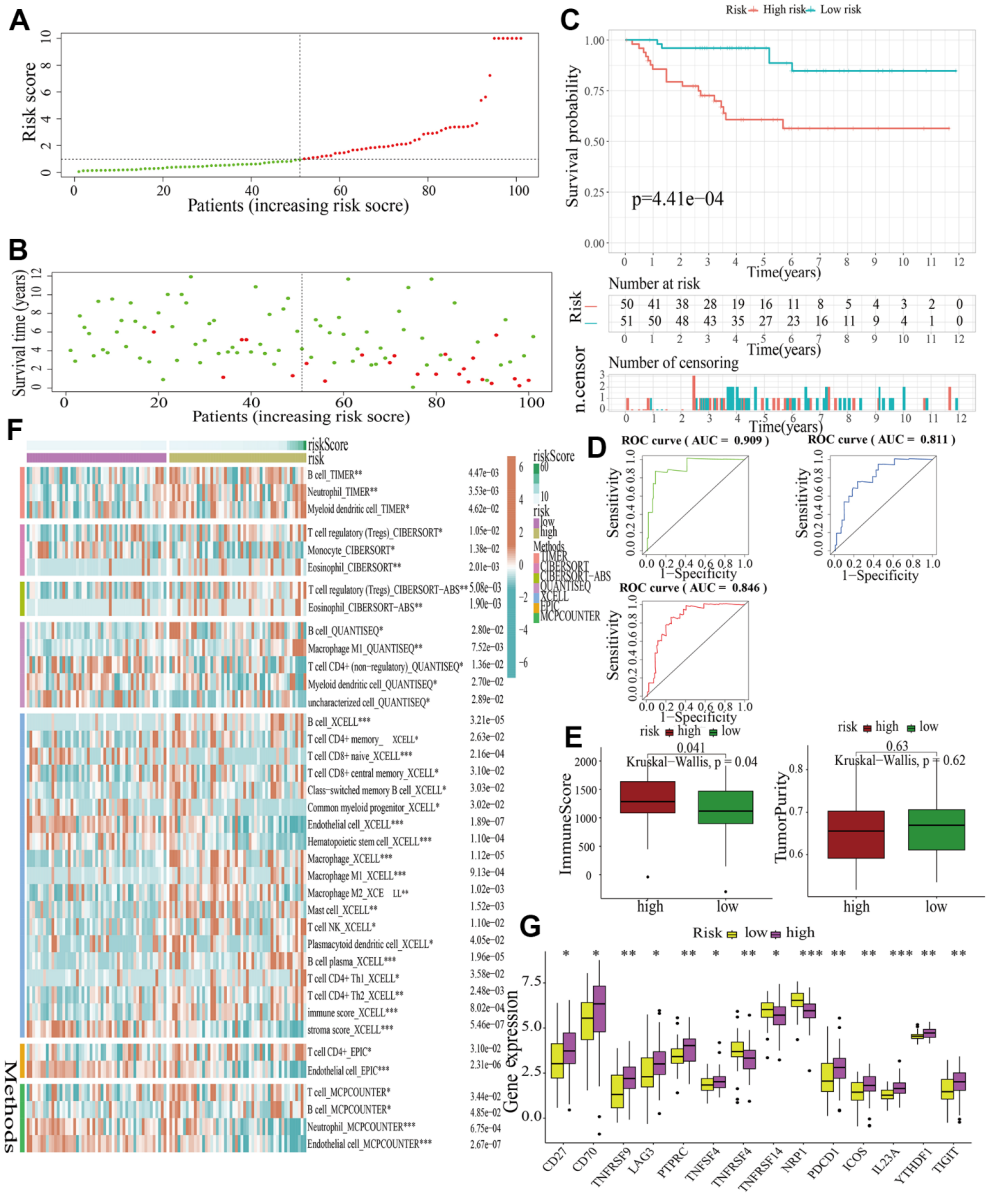
**External validation of a MAPK-related signature in test3 cohort.** (**A**) The distinguishment of high- and low-risk subgroups based on the median risk score in train cohort. (**B**) The distributions of the risk score and survival status. (**C**) Survival analysis in test3 cohort. (**D**) ROC curves of 1-, 3-, and 5-year survival. (**E**) Assessment of TME by “ESTIMATE”. (**F**) The discrepancies of immune response in high- and low-risk subgroups. (**G**) The discrepancies of ICD expression in high- and low-risk subgroups.

For further signature validation, all the analyses conducted above were performed in the test1, test2, and test3 cohorts. In the three test cohorts, similar results were obtained. First, risk scores were calculated and then all the samples were grouped into high- and low-risk subgroups in the three test cohorts respectively (Figures 6A, 7A, 8A). The distributions of the risk scores and survival status were showed in Figures 6B, 7B, 8B. The survival analysis revealed that samples in the high-risk subgroup were more likely to die (Figures 6C, 7C, 8C). The AUCs of the ROC curves also demonstrated the diagnostic value of the MAPKS: the AUCs had values of 0.672, 0.621, and 0.613 in test1 cohort, 0.719, 0.697, and 0.712 in test2 cohort, and 0.909, 0.811, and 0.846 in test3 cohort for 1-, 3-, and 5-year respectively (Figures 6D, 7D, 8D). Samples in the high-risk subgroup in the three cohorts also showed higher immune score but lower tumor purity (Figures 6E, 7E, 8E). As for the immune response, Treg had higher proportion while Endothelial cell had lower proportion in high-risk subgroup in the three test cohorts consistently (Figures 6F, 7F, 8F). The expression levels of ICGs in high- and low-risk subgroups were also compared and it was found that CD27, CD70, TNFRSF9, LAG3, PDCD1, ICOS, IL23A, and TIGIT up-regulated while NRP1 down-regulated in the high-risk subgroup in all three test cohorts (Figures 6G, 7G, 8G).

### The estimation of MAPK pathway and the expression of genes in the MAPKS in different celltypes

**Figure 9:**
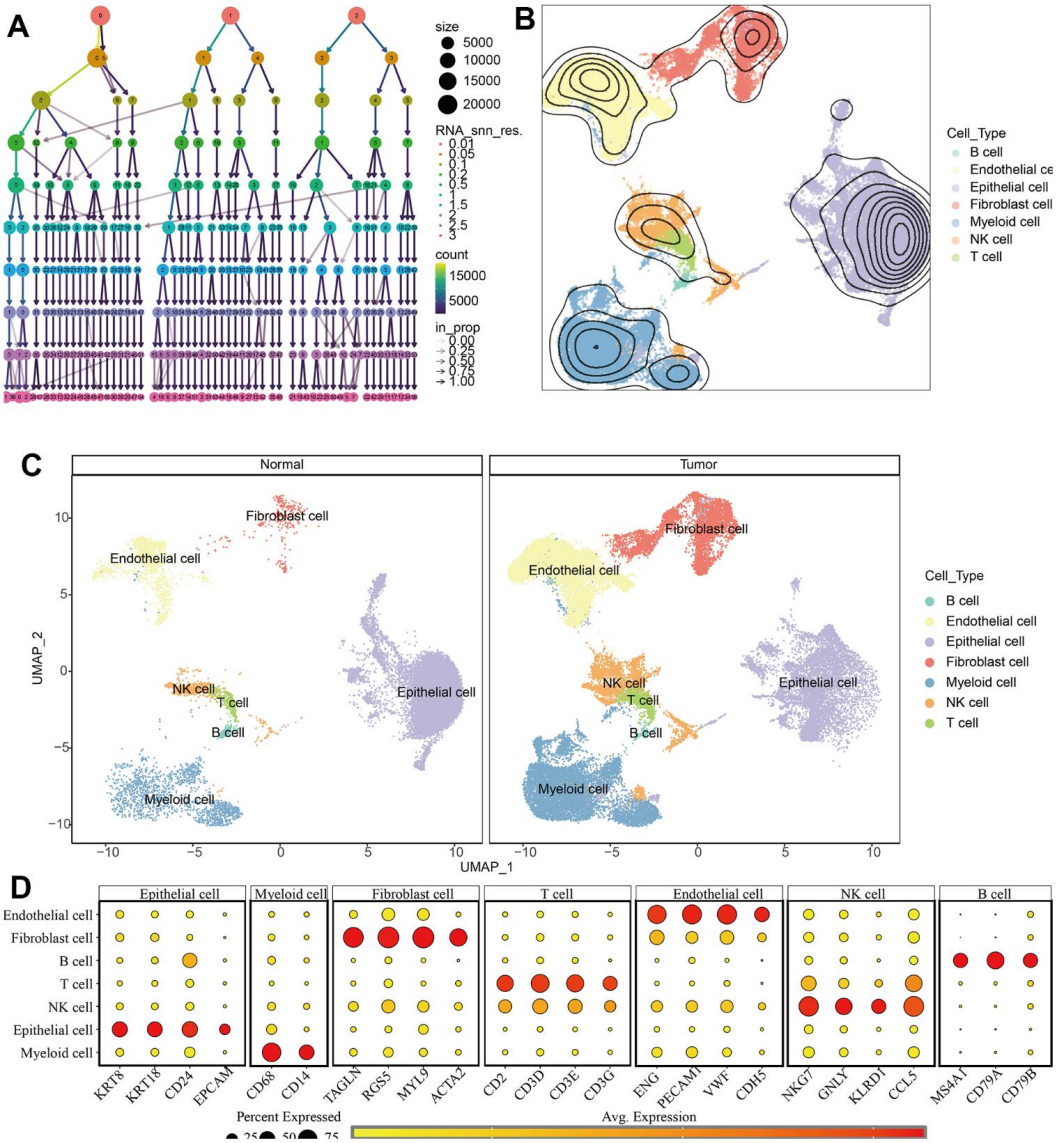
**Identification of different cell types in KIRC and normal samples.** (**A**) The clustree for identifying suitable cell clusters. (**B**) Different types of cells in all samples. (**C**) Different types of cells in KIRC and normal samples. (**D**) The expression of marker genes in each cell type.

**Figure 10:**
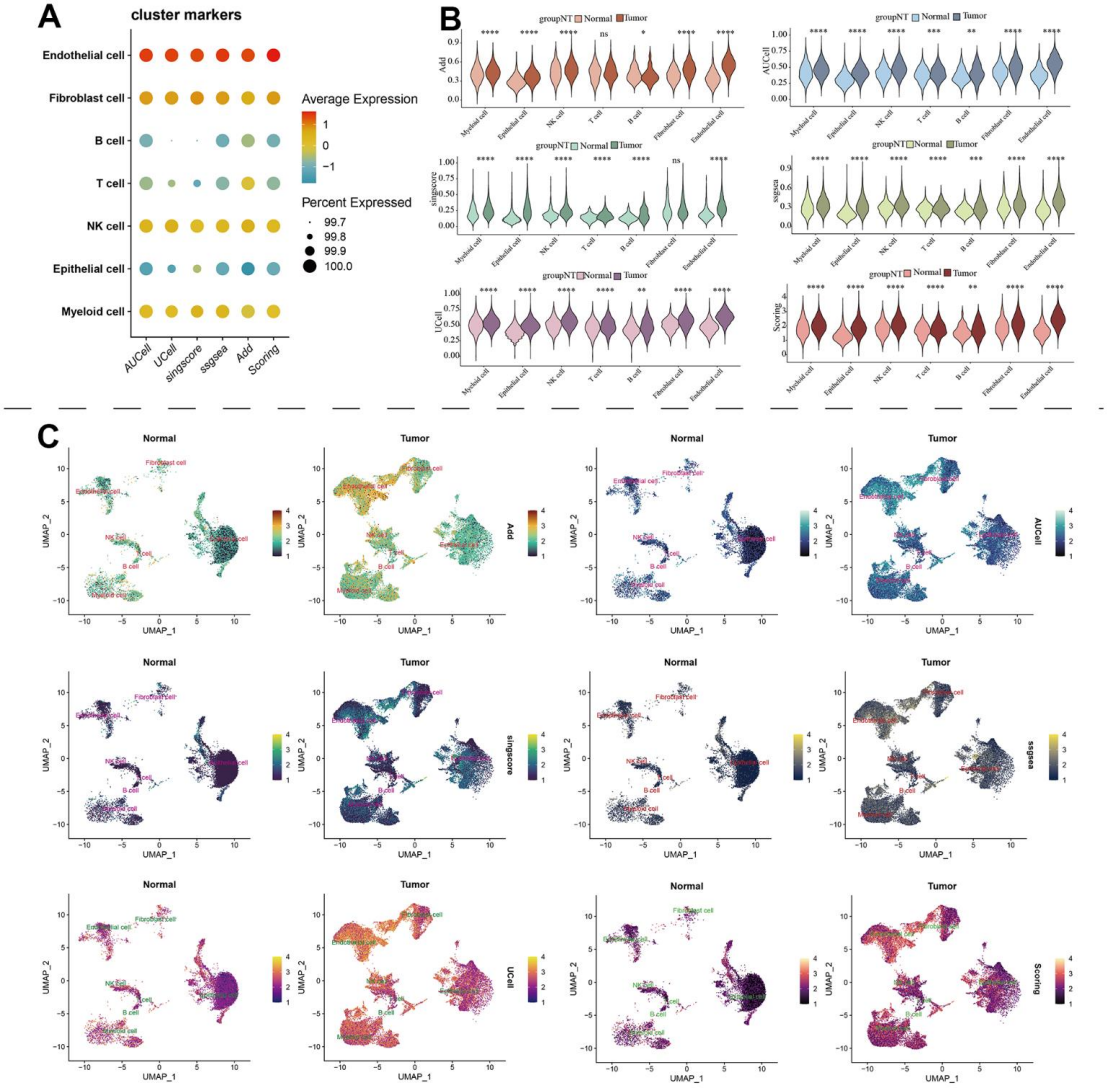
**The estimation of MAPK pathway based on scRNA-Seq data.** (**A**) The pathway score of MAPK signaling in each cell type; (**B**) The discrepancies of MAPK score in each cell type between KIRC and normal samples; (**C**) The detailed MAPK pathway activity shown in a UMAP plot.

The scRNA-seq data of 7 KIRC samples and 5 normal samples were integrated. After the quality control (Supplementary Figure 1), 56602 cells were grouped into 43 clusters (Figure 9A and Supplementary Figure 2A, 2B). Then these clusters were defined as different cell types based on the specific markers (Figure 9B–9D and Supplementary Figure 2C). The pathway score of MAPK signaling in different cell type was estimated. It suggested that the MAPK pathway was active in each celltype. Especially, it had superior score in endothelial cells (Figure 10A). After comparing the MAPK activity of each cell type between tumor samples and normal samples, we found that the statistical discrepancies existed in myeloid, epithelial, NK, B, and endothelial cells (Figure 10B, 10C).

### Identification and validation of hub genes in the occurrence of KIRC

**Figure 11:**
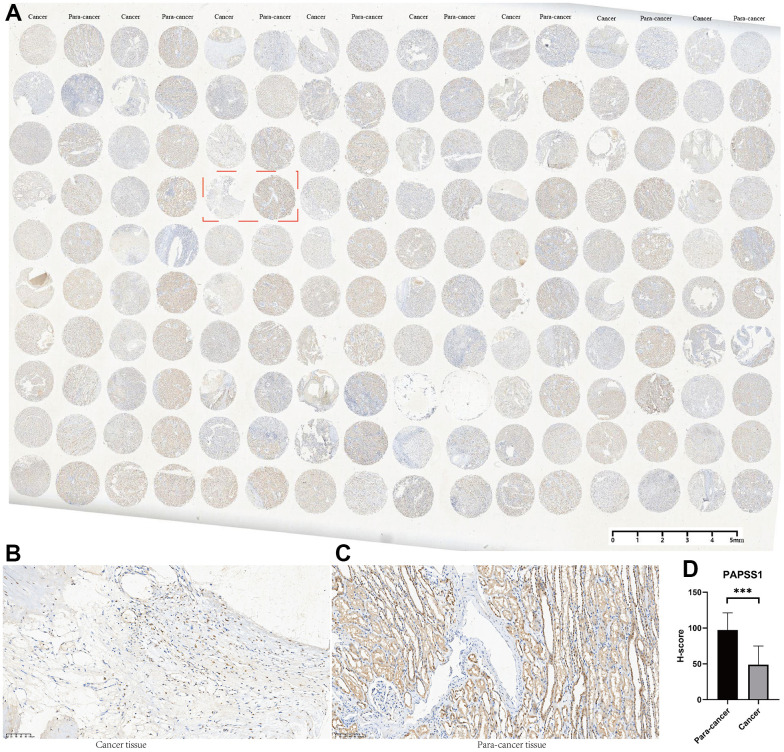
**The tissue microarray and immunohistochemistry (IHC) of PAPSS1.** (**A**) Immunohistochemical maps for all samples. (**B**) Immunohistochemical maps of typical cancer samples. (**C**) Immunohistochemical maps of typical para-cancer samples. (**D**) Immunohistochemical statistical analysis results.

Among all the 11 genes in the MAPKS, 3 genes showed statistically distinct mRNA expression between KIRC and normal samples. Compared with normal samples, the mRNA levels of MAP3K11 and SPRED1 increased while the mRNA level of PAPSS1 decreased in KIRC (Supplementary Figure 3A). In addition, the expression levels of the three genes were lower in advanced KIRC (Supplementary Figure 3B, 3C). Then the tissue microarray and immunohistochemistry (IHC) was performed to demonstrate the expression level of these three genes. And the PAPSS1, MAP3K11, and SPRED1 showed lower IHC scores in KIRC compared with para-cancer samples (*p*<0.05) (Figure 11 and Supplementary Figures 4, 5).

## DISCUSSION

As the major subtype of RCC, KIRC is characterized with high heterogeneity and poor prognosis [24–26]. Due to the profound influence of MAPK signaling pathway on the metabolism and progression of cancer [27–31], comprehensive alterations of MAPK-related genes in pan-cancer need to be summarized. In view of the interactions between pathways [32, 33], the alteration of MAPK pathway activity may affect various pathways in cancer. In the study, the pan-cancer analyses about the influence of MAPK pathway revealed that the expression of many MAPK-related genes varied with the occurrence and development of cancers.

Considering the significant role of MAPK pathway in pan-cancer and the rare reports about the relationship between MAPK pathway and KIRC, we focused on the alterations of MAPK pathway and corresponding influence in KIRC. Based on the expression of the MAPK genes, KIRC samples were group into three clusters with different MAPK signaling activity. In the in-depth research, it was revealed that MAPK-active samples had higher survival rate while MAPK-inactive samples had worse survival. With different status of MAPK signaling, metabolism pathways and immune pathways showed different activities in these three clusters. MAPK signaling also showed influence on the status of various cell deaths including curroptosis, immunogenic cell death, necroptosis, apoptosis, autophagy, ferropotosis, phagocytosis, necrosis, pyroptosis, PANoptosis, and disulfidptosis. TME and immune response also differed in the three clusters. Most immune cells except Tfh had positive correlation with MAPK activity. Of note, the responses of mast cells, Treg, type-II-IFN, and neutrophil were positively related to the MAPK activity.

As for drug therapy, samples with different MAPK activity might have different drug sensitivity. MAPK-active samples might be sensitive to Irinotecan and Topotecan, while MAPK-inactive samples may be sensitive to Cisplatin, Cyclophosphamide, Cytarabine, Docetaxel, Gefitinib, Lapatinib, Nilotinib, Sorafenib, Temozolomide, and Vinblastine.

In view of the influence of MAPK pathway activity on KIRC in multiple respects especially survival, the following analyses aimed to identify a signature for distinguish KIRC samples with distinct prognosis and microenvironment. Among all the genes related to MAPK pathway, 11 genes (i.e., SPRED3, ACTB, ARAF, MAP3K11, PAPSS1, TLN1, CALM1, AGK, MAP2K2, MAPK1, SPRED1) were identified as hub genes and used to construct a MAPKS. Compared with normal samples, the mRNA levels of MAP3K11 and SPRED1 increased while the mRNA level of PAPSS1 decreased in KIRC. The following IHC indicated that the PAPSS1, MAP3K11, and SPRED1 showed lower IHC score in KIRC compared with para-cancer samples. The post-transcriptional modifications play an important role in the overall regulation of gene expression, which might be the reason of the inconsistent changes in protein and mRNA [34, 35].

As for all the 11 genes in MAPSK, ACTB, AGK, MAP2K2, and MAPK1 were reported to regulate RCC. A single-cell analysis reveals ACTB is involved in the regulation of RCC metastasis and progression [36]. Also, it is demonstrated as an optimal reference gene in RCC by reverse transcription PCR (RT-PCR) [37]. AGK is found to promote RCC progression via activating the PI3K/AKT/GSK3β signalling pathway [38]. MAP2K2, another regulator of RCC, can promote its progression by affecting transcriptional activation of the MAP2K2-dependent ERK pathway [39]. MAPK1, one of the members of MAPK family, promotes RCC metastasis through HCP5/miR-214-3p/MAPK1 axis [40]. SPRED1, as a negative regulator of the MAPK pathway [41], influences tumor growth and metastasis in breast cancer. Also, the overexpression of SPRED1 can inhibit the proliferation, migration and invasion of HCC [42]. SPRED3, is reported to have an influence on EGFR mutated NSCLC [43], glioblastoma [44], and cervical carcinoma [45]. ARAF, is reported to be an oncogene in gallbladder cancer [46]. Its mutations imply resistance to the RAF inhibitor belvarafenib in melanoma [47]. In addition, mutant ARAF is found to be an oncogenic driver in lung adenocarcinoma and can be used as an indicator of sorafenib response [48]. MAP3K11 acts as a tumor suppressor [49] and a driver of cancer cachexia [50]. Also, it can regulate the malignancy of oral squamous cell carcinoma through facilitating autophagy [51]. Through regulating MAP3K11, NSCLC can be suppressed [52]. PAPSS1 is demonstrated as a suppressor gene in esophageal squamous cancer [53]. Out of this, it is reported to be associated with breast tumors previously [54]. TLN1, locating in focal adhesion, can regulate integrin signaling and promote cancer metastasis [55–58]. It can affect cell proliferation and differentiation in acute myeloid leukemia [59], define the risk of aggressive oral squamous cell carcinoma [60], and act as a regulator to suppress ovarian serous carcinoma [61]. CALM1 can engage in the formation of calmodulin and regulate proliferation, motility and differentiation through participating in signaling pathways [62]. Studies revealed that the expression of CALM1 was significantly linked to many types of cancer, such as prostate cancer [63], bladder cancer [64], and nasopharyngeal carcinoma [65].

Based on the MAPKS, the risk scores of all KIRC samples were calculated and two subgroups (i.e., high-risk subgroup and low-risk subgroup) were obtained in accordance with the risk score of the train cohort. The MAPKS helped to differentiate high-risk samples characterized by lower survival rates, higher immune scores, and reduced tumor purities. Of note, the elevated presence of Treg cells and the atypical expression of immune checkpoint molecules could potentially contribute to its adverse prognosis. As is reported, Treg cells can suppress effective tumor immunity. It was found that increasing infiltration of Treg cells was linked to poor prognosis of patients with tumors [66, 67]. In addition, tumor cells could disguise themselves as common components of the human body through immune checkpoint pathways [68]. As a consequence, the up-regulation of most ICGs in high-risk subgroup might account for the poor prognosis. All the discrepancies might be the potential therapy targets in KIRC and all the results above were demonstrated in the three test cohorts. In addition, the single-cell RNA sequencing (scRNA-seq) analysis of KIRC samples identified seven distinct cell types, which include B cells, myeloid cells, endothelial cells, NK cells, epithelial cells, T cells, and fibroblast cells. Endothelial cells in tumor tissues had obviously higher MAPK scores than normal tissues.

It is no doubt that a great signature MAPKS was constructed successfully. But there are still some limitations that need to be considered. First, the MAPKS was constructed utilizing a small number of KIRC samples from the TCGA and ArrayExpress databases. Then the results were obtained by utilizing bioinformatics researches. In the future, a large number of clinical samples need to be involved and fundamental experiments are necessary to utilize for further demonstration.

## CONCLUSIONS

With the development of tumors, MAPK pathway altered significantly in pan-cancer. Especially in KIRC, the status of MAPK pathway linked to the survival rate and drug sensitivity. The MAPKS was successfully constructed and demonstrated to help distinguish KIRC samples into different subgroups with distinct prognosis and tumor microenvironment. All the findings will contribute to the individualized treatment in KIRC.

## Supplementary Material

Supplementary Figures
