# Supplementary material for: Combination of bulk RNA sequencing and scRNA sequencing uncover the molecular characteristics of MAPK signaling in kidney renal clear cell carcinoma
Source: Aging (Albany NY). 2024 Jan 12;16(2):1414–39. doi: 10.18632/aging.205436 (PMC10866414; doi:10.18632/aging.205436)
Supplement: Supplementary Figures [file aging-16-205436-s001.pdf]

SUPPLEMENTARY FIGURES

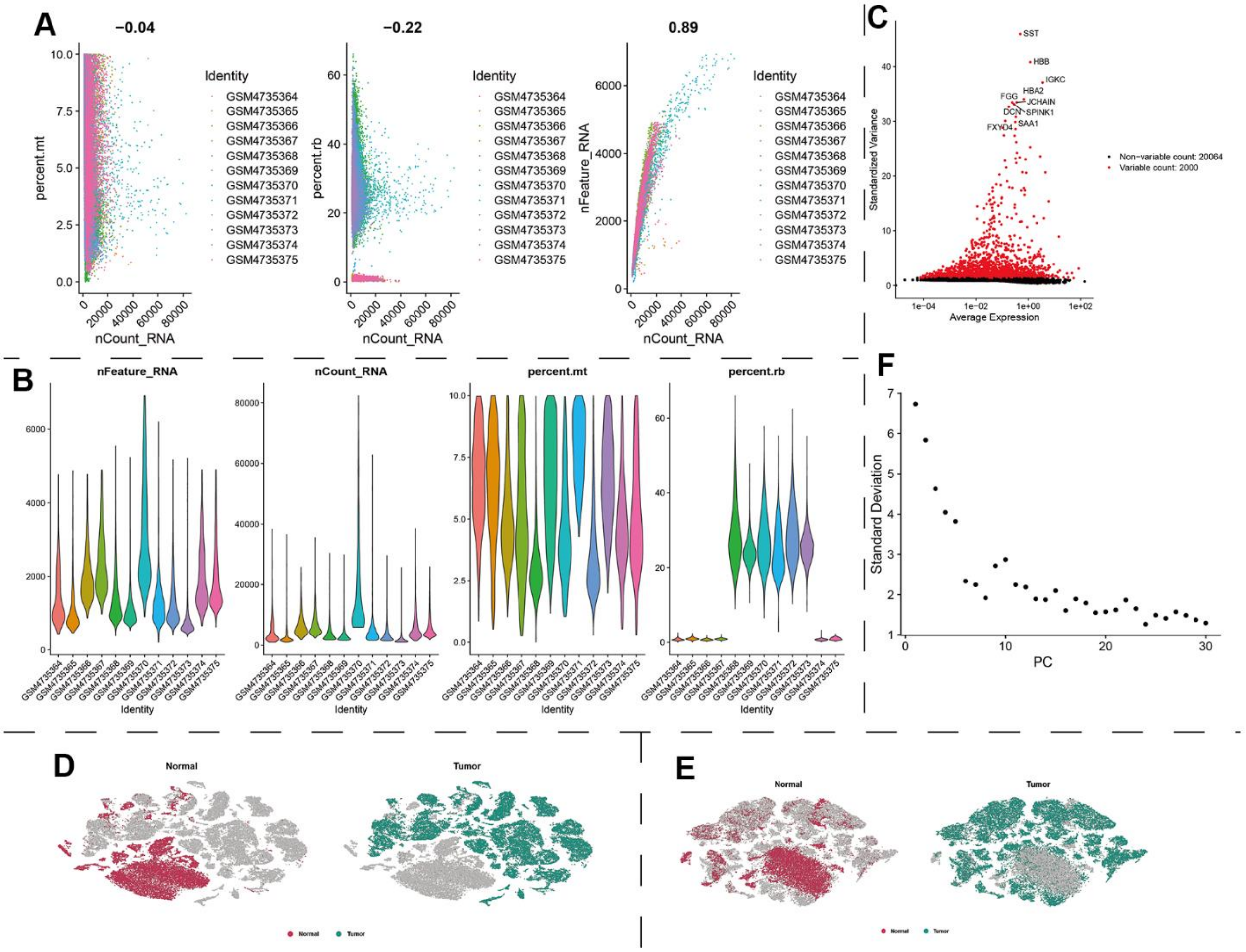

Supplementary Figure 1. Single-cell data processing. (**A**, **B**) Data quality control of sc-RNA data. (**C**) Highly variable genes of the data. (**D**, **E**) Cell distribution before and after removing the batch effect by Harmony. (**F**) ElbowPlot for identifying suitable PC number.

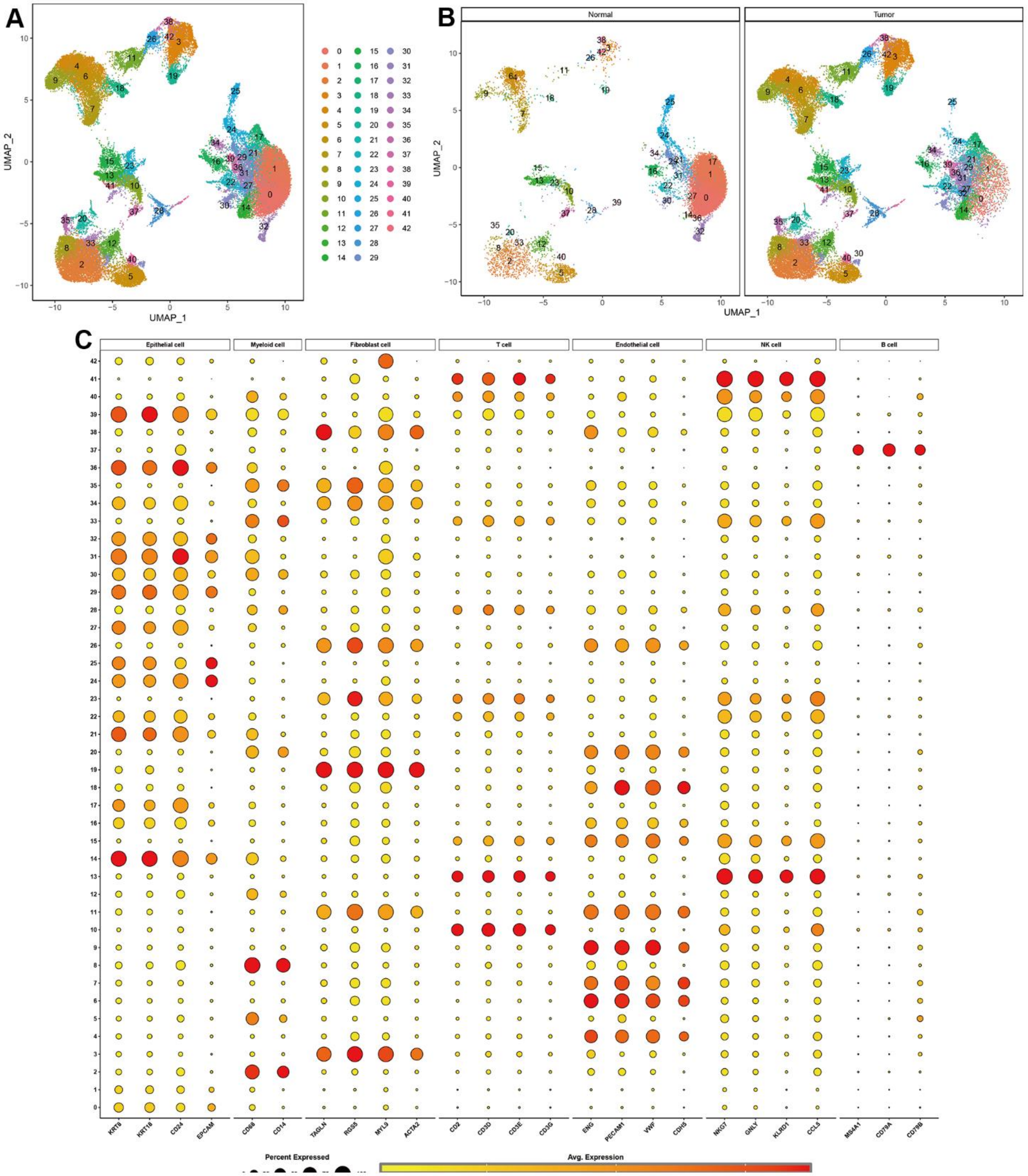

Supplementary Figure 2. Single-cell dimension reduction and annotation process. (**A**) UMAP dimension reduction plots for all samples. (**B**) UMAP dimension reduction plots for normal and tumor groups. (**C**) Single-cell annotation process.

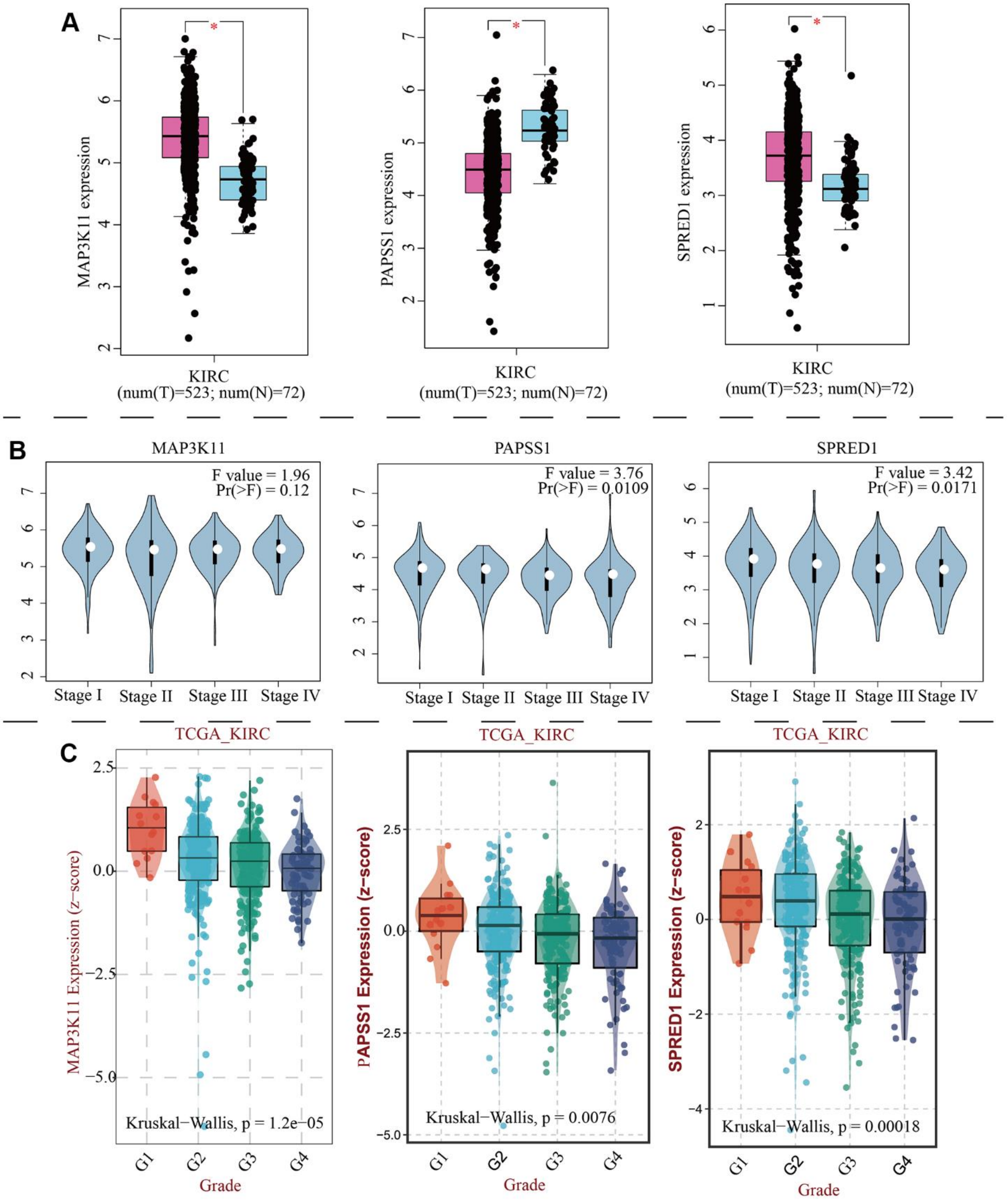

Supplementary Figure 3. Identification of hub genes in the occurrence of KIRC. (**A**) The mRNA expression of hub genes. (**B**) The relationship between the expression of each hub gene and tumor stage in KIRC. (**C**) The relationship between the expression of each hub gene and tumor grade in KIRC.

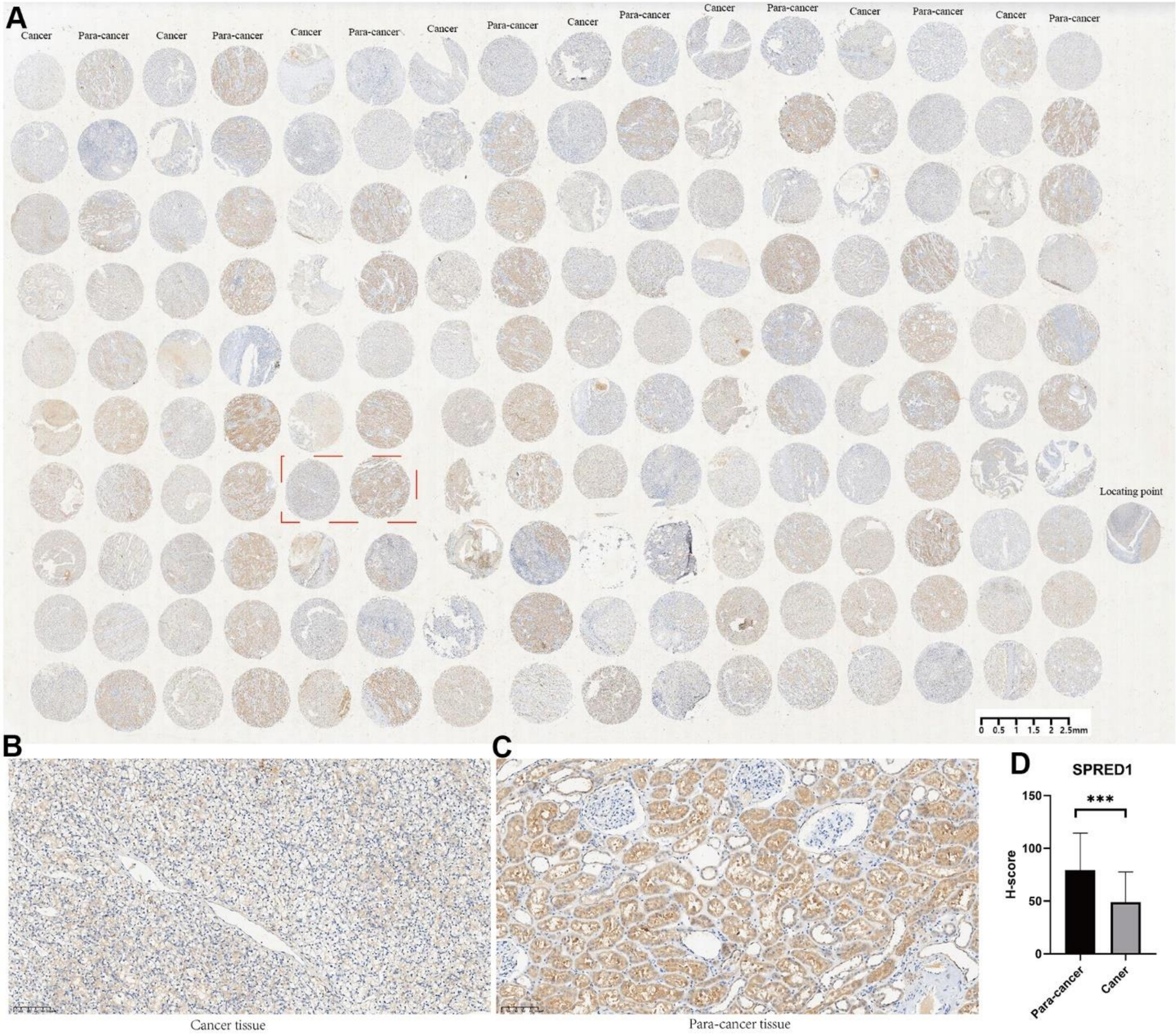

Supplementary Figure 4. The tissue microarray and immunohistochemistry (IHC) of SPRED1. (**A**) Immunohistochemical maps for all samples. (**B**) Immunohistochemical maps of typical cancer samples. (**C**) Immunohistochemical maps of typical para-cancer samples. (**D**) Immunohistochemical statistical analysis results.

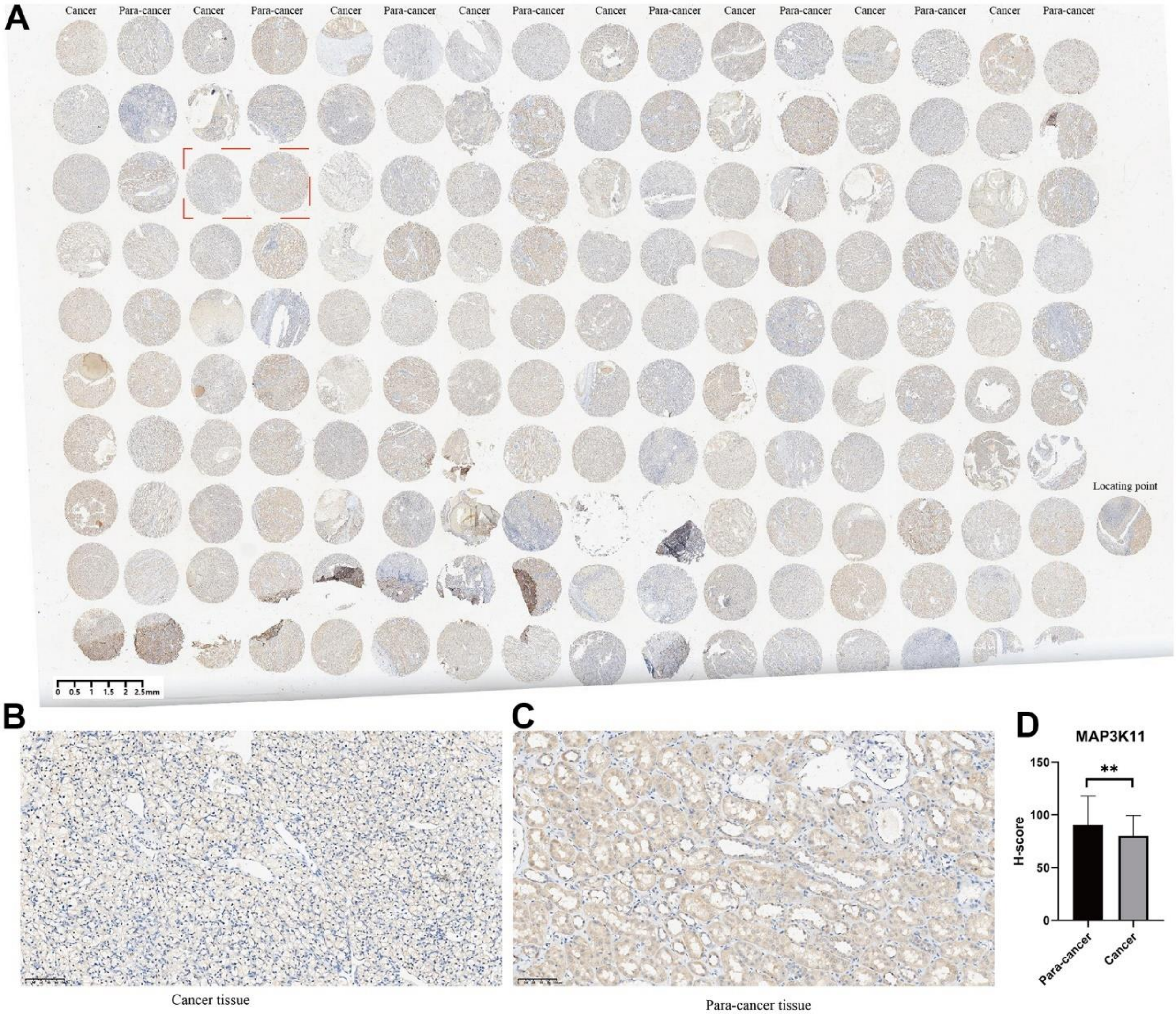

Supplementary Figure 5. The tissue microarray and immunohistochemistry (IHC) of MAP3K11. (**A**) Immunohistochemical maps for all samples. (**B**) Immunohistochemical maps of typical cancer samples. (**C**) Immunohistochemical maps of typical para-cancer samples. (**D**) Immunohistochemical statistical analysis results.
